# Multi-Objective Optimization of Low-Velocity Impact and Compression Behavior of 3D-Printed PLA Cubic Samples

**DOI:** 10.3390/polym17050627

**Published:** 2025-02-26

**Authors:** Oguz Dogan, Muhammed S. Kamer, Mehmet F. Sahan

**Affiliations:** 1 Department of Mechanical Engineering, Faculty of Engineering and Architecture, Kahramanmaras Sutcu Imam University, Kahramanmaras 46040, Turkey; msafakamer@ksu.edu.tr; 2 Department of Civil Engineering, Faculty of Engineering, Adiyaman University, Adiyaman 02040, Turkey; mfs@adiyaman.edu.tr

**Keywords:** compression, low-velocity impact, 3D printing, additive manufacturing, multi-objective optimization

## Abstract

This study investigates how various 3D printing parameters influence mechanical properties, specifically strength in compression and low-velocity impact (LVI) tests, and identifies the best printing parameters (layer thickness, nozzle diameter, and infill density) that lead to durable samples. Utilizing a Taguchi L_9_ orthogonal array, the study systematically examined the effects of three critical 3D printing parameters on the mechanical strength of cubic test samples. Nine experimental configurations were tested, each subjected to compression and LVI tests according to ASTM standards. Statistical analyses, including analysis of variance (ANOVA) and grey relational analysis (GRA), were employed to evaluate parameter significance and optimize results. Infill density significantly influenced the compression tests, while nozzle diameter was the most impactful parameter in LVI tests. Layer thickness had a minimal influence on both outcomes. Additionally, applying GRA revealed that optimal 3D printing parameters differ when considering the two mechanical properties simultaneously, highlighting the complexity of achieving balanced performance in 3D-printed structures. The application of the Taguchi method to optimize 3D printing parameters improved the mechanical properties of printed materials while significantly reducing the number of required experiments. By employing an efficient experimental design, this research demonstrates how to achieve high-quality results in compression and LVI tests with minimal resource use and time investment. Additionally, integrating GRA for the simultaneous optimization of multiple performance characteristics further enhances the practical applicability of the findings in additive manufacturing.

## 1. Introduction

Unlike conventional manufacturing methods, additive manufacturing (AM) is an innovative technique that allows products to be produced by adding materials layer by layer. AM minimizes material waste, enables the production of complex geometries, and offers flexibility and faster prototyping. Due to these advantages, additive manufacturing is used in various applications, from automotive to aviation, medical device, and jewelry production [1]. There are many different methods of additive manufacturing, such as laminated object manufacturing (LOM), selective laser sintering (SLS), laser-engineered net shaping (LENS), fused deposition modeling (FDM), etc. FDM is one of the most commonly used methods. The first material in the form of a filament is brought a few degrees above its melting temperature and turned into liquid in the FDM method. Afterward, the liquid material is continuously placed layer by layer on a table by passing through the nozzle [2]. The FDM method has many advantages, such as geometric independence, simple operation, low cost, fast manufacturing, and the ability to combine different materials. The production availability of the FDM method has increased dramatically with the widespread use of 3D printers [3]. Since products produced with 3D printers are frequently used, many researchers have performed much work on determining the mechanical properties of products produced. The tensile [4], compression [5], bending and damping [6], impact [7], fatigue [8,9], and creep [10] properties of products produced by 3D printing are investigated in different studies. In addition, the finite element method is also used to determine the mechanical properties of the products produced with the FDM method [11,12].

Production using the FDM method with a 3D printer provides manufacturers with full-filling or infill pattern options. Full filling requires more material, time, and money. On the other hand, using the infill pattern provides high energy absorption, a high strength–mass ratio, and high rigidity [13]. Thus, structures produced with infill patterns are used as the core material of many designs. When the literature is examined, it is seen that these structures are mainly exposed to compression and impact loads. Nace et al. [14] investigated the compression behavior of 3D-printed infill patterns, which included concentric, cross, cross 3D, and gyroid. Cross and cross 3D were the internal structures with the highest compressive strength. Qin et al. [15] produced PLA-based composite filaments using an extruder machine. Cellular test samples with different lattice structures were made of these novel filaments using a 3D printer. The compression and deformation performances of these structures were experimentally examined. Silva et al. [16] defined the effects of geometric and manufacturing parameters on the compression behavior of the 3D-printed cubic test samples. The influence of cell size, cell shape, strut diameter, feedstock, and layer height were investigated. As a result of the compression tests, cell geometry was defined as the parameter with the largest effect on mechanical properties. The influence of feedstock and layer thickness was described as minor. Kamer and Dogan [17] investigated the effects of infill pattern and compression axis on the compression behavior of 3D-printed cubic test samples. A core material was selected for octet, grid, cubic, quarter-cubic, and gyroid infill patterns. According to the compression test, the octet, cubic, and quarter-cubic structures had similar strengths for all axes. On the other hand, test samples with a grid internal structure had the highest strength on the z-axis, while the x- and y-axes had the lowest. Ma et al. [18] researched the effects of material type, infill pattern, and density on the compressive strength of 3D-printed cubic samples. Based on the experimental study, the infill pattern, density, and material type greatly affected the compressive and crashworthiness characteristics of the 3D-printed test samples. The effects of four different infill patterns (triangle, grid, quarter cubic, tri-hexagon) on the LVI and compression properties of 3D-printed PLA samples were examined by Aloyaydi et al. [19]. According to the test results, test samples with a triangular infill pattern absorbed the most energy because they had more sheared/contact layers than the other patterns. Smardzewski et al. [20] determined the compression and LVI properties of wood-based sandwich panels. Further compression and LVI tests were conducted by Ye et al. [21]. In the tests, 3D-printed CCFR self-sensing honeycomb structures were used. As a result of the experimental study, the 3D-printed CCFR self-sensing honeycomb structures performed well in LVI tests, CAI tests, and in situ structural health monitoring. Kaveloglu et al. [22]. investigated the LVI behaviors of sandwich composites. The core material of the sandwich composites was produced with a 3D printer using PLA material. The impact strengths of sandwich structures with three different cell widths were between 80J and 100J.

Three-dimensional printers allow users to change many production parameters during the production phase. The main 3D production parameters are nozzle diameter, layer height, infill type and density, table and nozzle temperature, printing speed, raster angle, etc. By changing these parameters, the mechanical properties of the product can be changed to the desired extent. In addition, optimum designs can be achieved by examining the effects of these parameters. For this reason, many researchers have experimentally examined the effects of 3D printer production parameters on the mechanical properties of the product. Wang et al. [23] investigated the effects of process parameters on the dynamic mechanical properties and tensile strength of 3D-printed PLA materials. The effects of the printing angle, layer thickness, fill rate, and nozzle temperature on the elastic modulus, tensile strength, elongation at break, storage modulus, loss modulus, and loss factor were investigated deeply. It was found that each process parameter had great effects on the examined mechanical properties. The effects of printing speed and temperature on the mechanical properties of 3D-printed PLA test samples were investigated by Tang et al. [24]. When the printing temperature increased, the tensile strength first rose and then decreased. On the other hand, the mechanical properties increased with an increase in printing speed. Chacóna et al. [25] investigated the effects of the process parameters (build orientation, layer thickness, feed rate) on tensile and flexural strength experimentally. The effect of each parameter varied from case to case. Thus, the interaction of each parameter examined was explained in detail. Hsueh et al. [26] examined the effects of printing temperature and speed on the tensile, compression, bending strength, and thermal characteristics of 3D-printed PLA PETG samples. As a result of the experimental study, the mechanical properties of the PLA test samples increased when the printing temperature and speed increased. However, the mechanical properties of the PETG material decreased with the increase in the printing speed. The effects of different process parameters on the tensile and compressive strength of 3D-printed PLA and ABS were investigated by Farazin et al. [27] and Abbas et al. [28], respectively. Rajpurohit et al. [29] examined the effects of raster angle, layer height, and raster width on the absorbed impact energy of 3D-printed PLA test samples. The raster angle was the most influential parameter on the impact energy. However, raster width and layer height were not statistically significant. Irez et al. [30] investigated the effects of layer thickness and printing speed on the absorbed impact energy of 3D-printed test samples. When the layer thickness was increased from 0.16 to 0.28 mm, the absorbed impact energy decreased by approximately 37%. In parallel with these results, absorbed energy decreased by 37% upon increasing the printing speed from 50 mm/min to 70 mm/min.

When the literature is examined, it is seen that there are many studies on the examination of the mechanical properties of test samples produced in 3D printers with the FDM method. These studies generally determine mechanical properties such as tensile strength, compression, impact, fatigue, creep, etc. In addition, many studies examine the effects of 3D printer process parameters on these mechanical properties. This study investigated the effects of various 3D printing parameters on the mechanical properties of printed cubic structures. By employing the Taguchi method, ANOVA, and GRA, we systematically identified and optimized the significant parameters influencing these mechanical properties. The results revealed that infill density emerged as the most impactful parameter for enhancing the compressive strength of the samples, while nozzle diameter was found to be the most critical factor affecting the impact resistance. Notably, these findings underscore the complexity of the interactions between different printing parameters, as the optimal settings for compression did not align with those for LVI tests. The application of statistical methods, such as ANOVA and GRA, facilitated a comprehensive understanding of the parameters’ effects and enabled the simultaneous optimization of both maximum force per mass (MFPM) and absorbed energy per mass (AEPM). The GRA results indicated that a combination of parameters could yield superior performance across both mechanical properties, thereby providing a balanced approach for practical applications in 3D printing.

## 2. Materials and Methods

Test samples were produced with an Ultimaker 2+ 3D printer (Ultimaker B.V., Ultrecht, The Netherlands), which has a 223 × 223 × 205 mm total printing volume and 12.5 μm, 12.5 μm, and 5 μm dimensional tolerances on the x-, y-, and z-axes, respectively. eSUN PLA+ (cold white, diameter 2.85 mm, Shenzhen Esun Industrial Co., Ltd., Shenzhen, China) filaments were used to produce the test samples. The compression and LVI test samples were produced in a climatic laboratory at a constant room temperature of 25 °C.

The compression and LVI test samples were designed as solid cubes within the dimensions of 50 × 50 × 20 mm on the x-, y-, and z-axes, respectively, according to ASTM C365-16 [31], using Solidworks 2022 CAD software. The designed CAD geometries were converted into a stereolithograph “.stl” file format and exported to the Ultimaker Cura 4.12.1 software. The 3D printer process parameters were defined in Ultimaker Cura 4.12.1. Layer thickness, nozzle diameter, and infill density were selected as variable printing parameters for both compression and LVI impact tests. The variable 3D printing parameters and their levels are shown in Table 1. Other 3D printer process parameters were taken as default fixed values defined in Ultimaker Cura 4.12.1. The CAD geometries were divided into slices, and the G codes required for the 3D printer were created using this software. The compression and LVI test samples were produced using the same G codes obtained with the desired process parameters in the 3D printer. In this way, the aim was to minimize the difference between the produced test samples.

Twenty-seven compression and impact tests are required for the full-factorial test design in this study, separately for each test. Considering that each experiment is repeated three times, the number of test samples to be produced will be eighty-one for the compression and impact tests, separately. In total, 162 test samples need to be produced for both tests. Since production with a 3D printer takes a long time, the Taguchi method was used in the design of the experiment in this study. Taguchi’s approach uses orthogonal arrays to systematically arrange performance parameters and their corresponding levels in the design of the experiment. The Taguchi method aims to determine product quality and procedures with a minimum number of experiments to save cost and time. Unlike the exhaustive factorial design, which explores all possible combinations, the Taguchi technique evaluates probabilistic pairs.

In this study, instead of factorial design, a time-consuming and expensive method, a Taguchi L_9_ orthogonal array is applied using Minitab 21.41 software. To analyze the effect of the 3D printing process parameters with the Taguchi method, only nine tests will be sufficient for each case. Thanks to the Taguchi method, a total of eighteen tests and fifty-four test samples were produced in this study. The Taguchi L_9_ experimental design for compression and LVI tests and production time for 1 sample are given in Table 2. When a sample is produced for each experiment, the 3D printer needs to work for approximately 1100 min. Since the experiments are repeated three times, this time becomes 3300 min. A total of 6600 min of continuous operation of the 3D printer is required for printing compression and LVI test samples. The time requirement for production is much longer when considering the cleaning of the 3D printer, warming up, calibrating the table and preparing it for the writing process, waiting for cooling after the writing process, etc., for each sample. Therefore, the production of a maximum of four samples was completed in one day. Approximately 13,200 min of 3D printer working time and eighteen days were saved by applying the Taguchi method in this study. In addition, by reducing the number of experiments, the electricity consumption and filament requirement for the 3D printer were significantly reduced.

After the production of the test samples, mass measurements were performed. A KERN PLS 6200-2A(KERN GmbH, Balingen, Germany) precision balance with a sensitivity of 0.01 g was used to measure the masses of the test samples. In this way, the MFPM and AEPM of the produced compression and LVI test samples could be calculated.

**Figure 1:**
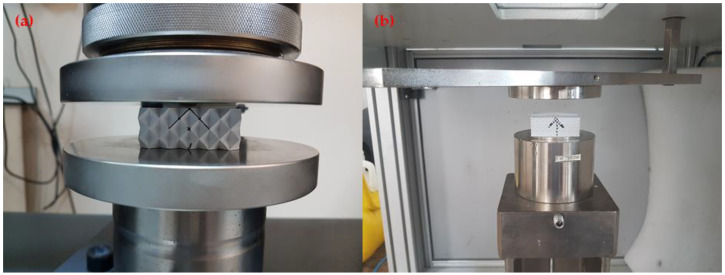
Experimental test setups: (**a**) compression test, (**b**) LVI test.

Compression tests were conducted following the standard [31]. The compression tests were performed using the compression side of a Zwick/Roell Z100 (ZwickRoell Group, Ulm, Germany) tensile testing device, which has 100 kN capacity (Figure 1a). The force applied during the tests was measured with a GTM brand K series 100 kN (GTM Testing and Metrology GmbH, Bickenbach, Germany) load cell, which has an associated measurement error of ±0.4%. The displacement was recorded using an AC servo motor system (Danaher Motion GmbH, Düsseldorf, Germany), which exhibited a positioning repeatability of ±2.0 μm. The compression speed was determined to be 0.5 mm/min [31]. The tests were repeated three times. In total, twenty-seven compression tests were completed. Force–displacement curves were obtained from the compression tests. MFPM values were used for the statistical analysis of the compressive tests. The MFPM was calculated using the following equation.

(1) $$\mathrm{MFPM}=\frac{\mathrm{Maximum}\;\mathrm{compressive}\;\mathrm{force}\;\left(N\right)}{\mathrm{Mass}\left(g\right)\;}$$

LVI tests were performed using the Instron Ceast 9350 LVI (Instron, Norwood, MA, USA) test device (Figure 1b) following the ASTM D7136-15 [32] standard. The impact tester operates according to the mass drop method, and the tests were carried out by freely dropping a 14.5 kg load onto the test samples. In the LVI tests, the energy was selected at a level where all samples were pierced entirely. To determine this level, preliminary experiments were carried out at different energies. In the preliminary tests, 125 J of impact energy completely pierced all the samples. Thus, it was determined how much energy each LVI sample absorbed. The pressure of the upper-pressure pneumatic pistons was set to 2 bar, and a 20 mm diameter spherical-tipped striker was used in the tests. The tests were repeated three times for each experiment. The AEPM values were calculated using the following equation:

(2) $$\mathrm{AEPM}=\frac{\mathrm{Absorbed}\;\mathrm{energy}\;\left(J\right)}{\mathrm{Mass}\left(g\right)}$$

The Taguchi method was subsequently applied to examine how various modeled parameters affected the solution to the problem. The Taguchi method calculates the signal-to-noise (S/N) ratios for each investigated factor [33]. It also calculates delta values based on these S/N ratios and establishes the rank of each factor. The Taguchi method converts outcomes from orthogonal experimental design into a signal-to-noise (S/N) ratio, measured in decibels (dB). The signal factor represents the actual value achieved in the system, while the noise factor refers to variables not included in the experimental setup that can influence the results. Noise sources encompass all factors that lead to deviations from the desired target value. The S/N ratio is defined in three distinct forms: “larger is better”, “lower is better”, and “nominal is better’’ [34]. The equations corresponding to these forms are provided below:

Larger is better:

(3) $$\frac{S}{N}=-10\log\left(\frac{1}{n}\overset{n}{\underset{n=1}{\sum }}\frac{1}{y_{i}^{2}}\right).$$

Lower is better:

(4) $$\frac{S}{N}=-10\log\left(\frac{1}{n}\overset{n}{\underset{n=1}{\sum }}y_{i}^{2}\right).$$

Nominal is better:

(5) $$\frac{S}{N}=10\log\left(\frac{{µ}^{2}}{\sigma^{2}}\right).$$

In this context, n denotes the number of experiments, μ represents the mean, σ indicates the standard deviation, and y_i_ refers to the data obtained from the i-th experiment [35].

The objective of this study was to identify the manufacturing parameters that achieve maximum strength in compression and LVI experiments. Consequently, the results of the experiments were analyzed using a method that computes the signal-to-noise ratio according to the “larger is better” criterion.

In this study, analysis of variance (ANOVA) is utilized to assess the individual impact of each 3D printing parameter on compression and LVI strength. This statistical technique analyzes experimental datasets to provide valuable insights. It is beneficial for evaluating the significance of the influence of parameters or their interactions on a particular response.

The steps involved in the ANOVA calculation are outlined as follows. First, the total sum of squares (SS_T_) was computed using the following equation [35].

(6) $${\mathrm{SS}}_{T}=\overset{n}{\underset{i=1}{\sum }}{\left(y_{i}-\;\bar{y}\right)}^{2}.$$

(7) $$\bar{y}=\frac{1}{n}\overset{n}{\underset{i=1}{\sum }}y_{i}.$$

Here, n represents the total number of cases in the orthogonal array, while y_i_ denotes the experimental result for the i-th experiment.

The sum of the squared deviations SSP can be calculated as follows:

(8) $${\mathrm{SS}}_{P}=\overset{t}{\underset{j=1}{\sum }}\frac{{\left({\mathrm{Sy}}_{j}\right)}^{2}}{t}-\frac{1}{n}{\left[\overset{n}{\underset{i=1}{\sum }}y_{i}\right]}^{2}.$$

In this context, P refers to one of the parameters, j indicates the level number associated with parameter P, and t represents the number of repetitions for each level of parameter P. Additionally, Sy_j_ is the sum of the experimental results related to parameter P at level j.

Finally, the percentage contribution *ρ* is determined as follows:

(9) $$\rho_{\rho }=\frac{{\mathrm{SS}}_{P}}{{\mathrm{SS}}_{T}}$$

The Taguchi and ANOVA methods are designed to analyze how varying parameters influence the solution to a single problem. Therefore, in this study, the GRA method is used to investigate the effects of the 3D printing parameters on the compression and LVI impact strength of the test samples simultaneously. The GRA method enables the transformation of an optimization problem with multiple performance characteristics into a single-objective optimization problem [36].

The GRA calculation consists of four steps: 1. normalizing the results for each experimental result; 2. calculating the grey relational coefficient (GRC); 3. determining the weight factors; and 4. calculating the grey relational grade (GRG).

The normalization of the results for each experimental result is defined in three distinct forms, like the S/N ratio calculation. These are “higher is better”, “lower is better”, and “nominal is better’’ assumptions.

This study adopts the “higher is better” approach, similar to that used in the Taguchi method.

(10) $$y_{i}\left(k\right)=\frac{x_{i}^{0}\left(k\right)-{\mathrm{minx}}_{i}^{0}\left(k\right)}{\max x_{i}^{0}\left(k\right)-\min x_{i}^{0}\left(k\right)}$$

where i = 1, …, p; k = 1, …, r; p = number of the cases; y_i_(k) denotes the normalized value of the grey relational generation; ${\mathrm{maxx}}_{i}^{0}\left(k\right)$ and ${\mathrm{minx}}_{i}^{0}\left(k\right)$ represent the maximum and minimum values of $x_{i}^{0}\left(k\right)$; and x^0^ defines the optimum value. The GRC is calculated after normalization.

(11) $$\xi i\left(k\right)=\frac{\Delta_{\min}+\;\varphi \Delta_{\max}\;}{\Delta_{0i}\left(k\right)+\varphi \Delta_{\max}}$$

(12) $$\Delta_{0i}\left(k\right)=y_{0}\left(k\right)-y_{i}\left(k\right)$$

(13) $$\Delta_{\max\;}=\max_{\forall \mathrm{ji}}\max_{\forall k}y_{0}\left(k\right)-y_{i}\left(k\right)\;$$

(14) $$\Delta_{\min\;}={\mathrm{mix}}_{\forall j\varepsilon i}\min_{\forall k}y_{0}\left(k\right)-y_{i}\left(k\right)\;$$

where Δ_0i_ represents the deviation value between y_0_(k) and y_i_(k). Here, y_0_(k) is the reference sequence, while y_i_(k) is the comparative sequence. Δ_max_ and Δ_min_ denote the maximum and minimum values of Δ_0i_, respectively. φ is the identification coefficient, constrained within the range of 0 < φ < 1. According to previous studies, φ is generally set as 0.5 [33,37].

The GRG can be calculated using the following equation:

(15) $$\gamma_{i}=\frac{1}{n}\overset{n}{\underset{k=!}{\sum }}w_{k}\xi_{i}\left(k\right)$$

In this context, n represents the number of tests, w_k_ denotes the normalized weight for the k-th performance characteristic, and $\gamma_{i}$ indicates the overall GRG. Accurately determining w_k_ for each performance characteristic is crucial. The GRG reflects the degree of correlation between the comparative sequence and the referential sequence. A GRG of 1 indicates that these values are identical.

Yuce [38] noted that numerous researchers have employed an equal weight of 0.5 when calculating the GRG for multiple responses. However, the weight factor was determined using the following expression in this study.

(16) $$w_{i}=\frac{\sum_{j=1}^{p}{\mathrm{Delta}}_{\mathrm{ij}}}{\sum_{i=1}^{m}\sum_{j=1}^{p}{\mathrm{Delta}}_{\mathrm{ij}}}$$

In this context, p refers to the number of parameters, Delta signifies the range of the S/N ratio, and m represents the total number of responses.

**Figure 2:**
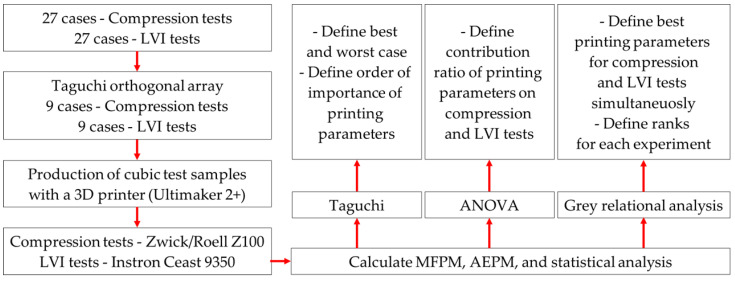
Flowchart of this study.

The methods used in this study are illustrated in Figure 2. Initially, the number of compression and LVI tests, which consisted of twenty-seven cases, was reduced to nine using the Taguchi orthogonal array. Then, cubic test samples were produced using a 3D printer based on the scenarios that were obtained. The manufactured test samples were subjected to compression and LVI tests using the Zwick/Roell Z100 and Instron Ceast 9350 testing machines, respectively. The data derived from the test results were utilized to calculate the MFPM and AEPM values. For the calculated values, the Taguchi method was applied again to determine the best and worst cases for each experiment individually. Additionally, the order of importance of the printing parameters was assessed. The contribution ratios of the printing parameters on compression and LVI tests were separately determined using the ANOVA method. Finally, the grey relation analysis method was employed to identify the optimal production parameters for both tests concurrently. Furthermore, the ranking of each experiment was also established using this method.

## 3. Results and Discussion

**Figure 3:**
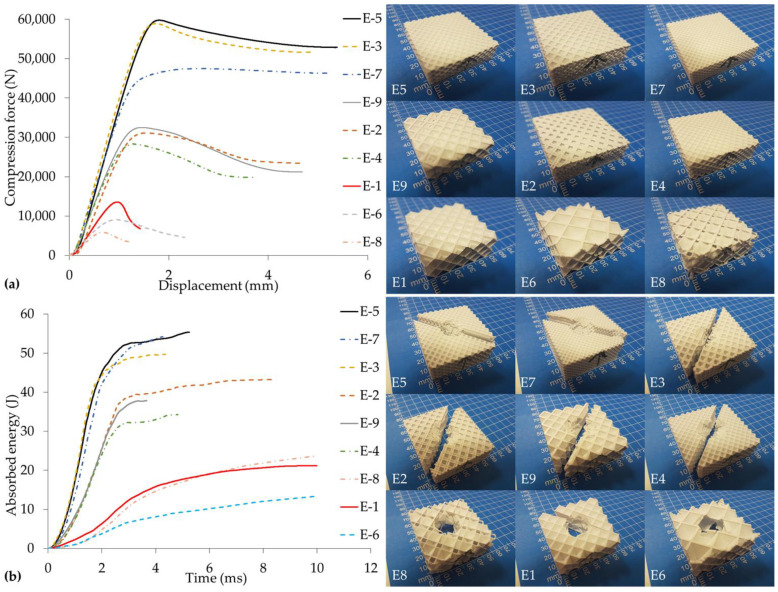
Test results and test sample damage images: (**a**) compression test force–displacement curves, (**b**) LVI test absorbed energy–time curves.

In this study, nine cases of compression and LVI were examined separately. The tests were repeated three times for each case. A total of fifty-four experiments were performed: twenty-seven compression tests and twenty-seven LVI tests. The results of the compression (a) and LVI (b) tests are given in Figure 3. Figure 3 was created using the true curves closest to the mean for each case. When the results are examined, it is seen that the maximum force and absorbed energy values are distributed in a very wide range. As the infill density increases, compression force and absorbed energy increase significantly. The effects of other 3D printer design parameters on compression force and absorbed energy cannot be directly inferred from Figure 3.

As the mass of the test samples increases, the compression force and absorbed energy values also increase. Therefore, to make the results independent of the mass, the MFPM and AEPM values were calculated using Equations (1) and (2). Test results, sample masses, and MFPM and AEPM values obtained from compression and LVI experiments are given in Table 3. After the mass-free process is performed, when the test results are examined, it cannot be directly seen which 3D printing parameter has the greatest impact on the results. Therefore, statistical methods were used to determine which parameter has the greatest effect on the test results.

A response table with S/N ratios for compressive and LVI tests is given in Table 4. The delta values were calculated, and the parameters were ranked based on the computed S/N ratios. According to the delta values, infill density is the most important printing parameter for the compression strength of the test sample. On the other hand, nozzle diameter is the most influential parameter in LVI tests. However, it is the least important parameter among the parameters examined for compressive strength. Layer thickness is the least impactful parameter on impact resistance and the second most influential parameter in compression tests.

**Figure 4:**
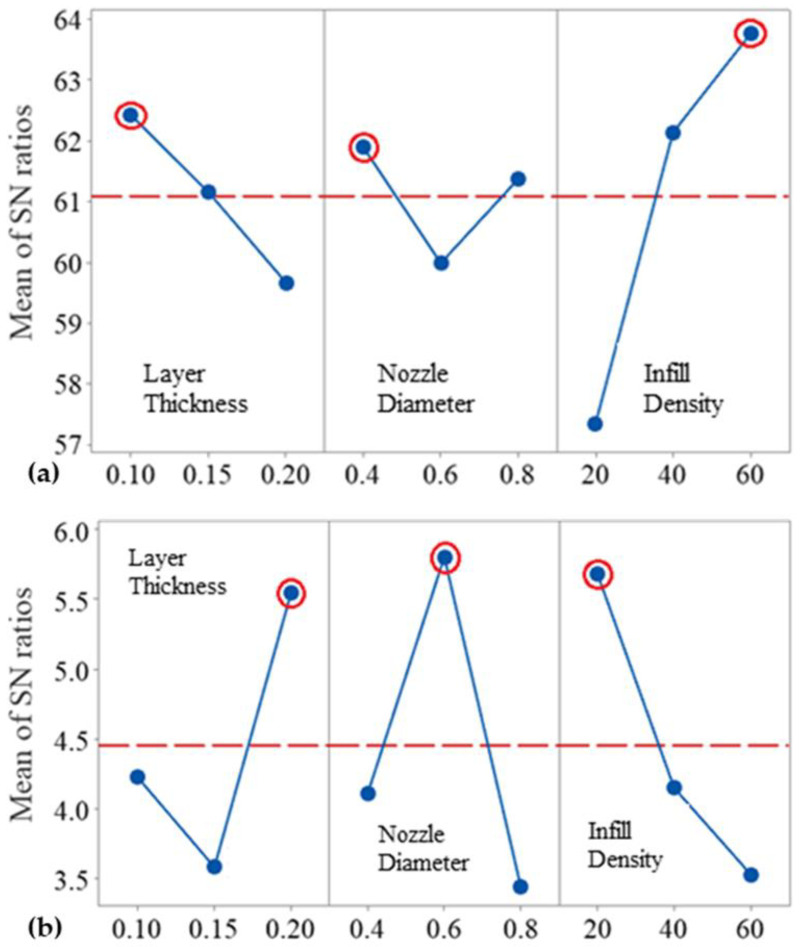
Optimum 3D printing parameters for (**a**) compression and (**b**) LVI tests.

The highest S/N ratio indicates the optimum value for the design parameter for a single test. The optimum 3D printing parameters for compression (a) and LVI (b) tests are marked with red circles in Figure 4. Values of 0.1 mm (A1) for layer thickness, 0.4 mm (B1) for nozzle diameter, and 60% (C3) for infill density are identified as the optimal 3D printing parameter values to achieve MFPM for compression tests. When Table 2 is examined, it is seen that the optimum printing parameters obtained for compression testing with the Taguchi method are not in the DOE. To verify this solution, new test samples were produced at the optimum printing parameters obtained by the Taguchi method and subjected to compression testing. According to the compression test, the optimal case is characterized by a maximum compression force of 58,185.14 N. Furthermore, the mass of the test sample was measured at 34.21 g. The MFPM for the optimal case during the compression test was calculated to be 1700.82 N/g. Upon reviewing Table 3, it is evident that the MFPM value exceeds that of all other cases analyzed. This indicates that the optimum result for a single output can be achieved using the Taguchi method without trying every possible scenario. It is also possible to identify the worst-case scenario for compressive tests by using Figure 4a. Values of 0.2 mm (A3) for layer thickness, 0.6 mm (B2) for nozzle diameter, and 20% (C1) for infill density are the worst-case 3D printing parameters for the compression tests. Experiment number E-8 represents these parameters according to the DOE in Table 2. The MFPM value of the compression test samples (E-8) produced with these parameters is 478.91 N/g (Illustrated in Table 3). This value is the lowest MFPM compared to the results of other compression tests. As the layer thickness increases, the MFPM value decreases. On the other hand, it increases with an increase in infill density. In addition, the MFPM exhibits a fluctuating change when the nozzle diameter is increased.

A 0.2 mm (A3) layer thickness, 0.6 mm (B2) nozzle diameter, and 20% (C1) infill density are seen as the optimal 3D printing parameter values to obtain a maximum AEPM value in LVI tests in Figure 4b. On the contrary, these parameters are the worst option for the compressive tests. As seen in the test result number E-8 in Table 3, the AEPM value is 2.67 J/g. This value is higher than all the other test results for LVI tests. The best 3D printing parameters were also determined using the Taguchi method without trying all experimental combinations for LVI tests. The 3D printing parameters of 0.15 mm (A2) for layer thickness, 0.8 mm (B3) for nozzle diameter, and 60% (C3) for infill density represent the worst case for the LVI test (Figure 4b). When Table 2 is examined, it is seen that this case is not among the experiments examined. Since this case was the worst option among LVI tests, no validation study was required. AEPM values decrease constantly as the infill density increases. However, as the layer thickness and nozzle diameter increase, AEPM values fluctuate.

The ANOVA results for the compression tests are presented in Table 5. Infill density is the most influential parameter affecting the MFPM values for these tests, with an effectiveness rate of 77.74%, consistent with the Taguchi results shown in Table 4. The second most significant parameter is layer thickness, with an impact rate of 9.26%. The nozzle diameter was found to have minimal effect on the compression tests.

The ANOVA results for the LVI tests can be found in Table 6. Nozzle diameter, the least important parameter for the MFPM, is the parameter with the most significant effect on the AEPM, with an effectiveness rate of 36.14%. The infill density is the second most important 3D printing parameter for the AEPM, with an impact rate of 30.86%. On the other hand, the layer thickness is the least impactful parameter for the AEPM. When the difference between the parameters affects the AEPM, it is seen that there is only a difference of about 10% between the most impactful parameter and the least impactful parameter. However, in compression tests, infill density has a much more significant effect than other parameters. These results show that both experimental groups exhibit different characteristics. Overall, the ANOVA and Taguchi results align well regarding the MFPM and AEPM values.

The effects of 3D printing parameters were examined in detail for each experimental group using Taguchi and ANOVA methods. However, using these methods, the best case cannot be determined for both experiments simultaneously. A multi-objective functional approach was employed to achieve the simultaneous optimization of both MFPM and AEPM, which is the objective in practical applications, and to explore their interrelations. MFPM and AEPM were combined into a single-objective function, referred to as multiple performance characteristics, utilizing GRA.

Assigning appropriate weight factors to each objective function is essential for achieving more accurate results in the GRA. Prior to determining the GRG, the weight factors for MFPM and AEPM were computed using Equation (16) and found to be 63.12% and 36.88%, respectively. These values are presented in Table 4. The GRG is calculated with the following equation.

(17) $$\mathrm{GRG}=0.6312GRC_{MFPM}+0.3688GRC_{AEPM}$$

The GRA results (normalized results, GRC, GRG, and ranks) are given in Table 7. When considering both MFPM and AEPM results, E-5 exhibits the highest GRG, indicating that it performs best among all the cases tested. The best 3D printing parameters are defined as 0.15 (A2) layer thickness, 0.6 (B2) nozzle diameter, and 60% (C3) infill density. According to Figure 4, the optimal solutions for the compression (MFPM) and LVI (AEPM) tests differ when analyzed separately and when evaluated together. This indicates that GRA analysis plays a crucial role in the suggested method of integrating different mechanical properties of 3D printed products with statistical techniques.

A response table for S/N ratios for the grey relation grade is given in Table 8. The delta values are calculated, and the 3D printing parameters are ranked according to the delta values. The infill density is the most influential parameter on both compression and LVI behaviors of the 3D printed cubic structures simultaneously. Nozzle diameter ranks as the second most important parameter, while layer thickness is considered the least impactful factor.

## 4. Conclusions

This study systematically examined the impact of significant 3D printing parameters (layer thickness, nozzle diameter, and infill density) on the mechanical properties of 3D-printed cubic structures, specifically focusing on MFPM during compression tests and AEPM during LVI tests. Utilizing a robust experimental design approach through the Taguchi method, we significantly narrowed down a comprehensive range of parameters, allowing for a focused analysis of their effects on mechanical performance. Additionally, the contribution rates of 3D printing parameters in compression and LVI tests were determined using the ANOVA method. Finally, using the GRA method, the 3D printing parameters that gave the best results simultaneously for compression and LVI impact tests were determined.

Infill density is the most important factor affecting the MFPM in compression tests. When the infill density increases, both the maximum compressive force and the MFPM values are increased significantly. According to the ANOVA results, the infill density contributes 77.74% of the MFPM in compression tests. This is because denser infill gives better material strength and resistance to compressive loads, which improves the overall strength of the printed samples. On the other hand, the infill density is determined as the second most important 3D printing parameter in the LVI impact tests, with a contribution rate of 30.86%. The nozzle diameter is very important for the AEPM during LVI tests, with a contribution rate of 36.14%. When the nozzle diameter increases, the energy absorption ability of the cubic test samples improves. However, the nozzle diameter does not affect compression strength. It is the least impactful parameter on the compression strength of the cubic test samples, with a contribution rate of 2.15%. This situation shows that different types of loads require different mechanical features. Layer thickness is the least important parameter for LVI tests and the second most important parameter for the compression tests, with contribution ratios of 25.9% and 9.26%, respectively. Thicker layers may weaken the bond between layers, leading to lower MFPM values. The best-performing experimental case (E-5) is determined using the GRA method for both compression and LVI tests simultaneously among the nine cases, which is decreased from twenty-seven cases using the orthogonal array. In addition, the infill density is determined as the most impactful 3D printing parameter in both the compression and LVI tests. On the other hand, layer thickness is defined as the least important according to the GRA results.

In conclusion, this study establishes a robust methodological framework for optimizing 3D printing parameters, emphasizing the necessity of a multifaceted approach to achieve the desired mechanical properties in printed structures. The combination of statistical analysis and practical experimentation paves the way for improved applications of 3D printing technology in various engineering domains.

## Figures and Tables

**Table 1:** Variable 3D printing parameters.

Parameters	Level 1	Level 2	Level 3
(A) Layer thickness (mm)	0.1	0.15	0.2
(B) Nozzle diameter (mm)	0.4	0.6	0.8
(C) Infill density (%)	20	40	60

**Table 2:** Taguchi L_9_ experimental design (DOE) for compression and LVI tests and production times.

Experiment Number	Layer Thickness (mm)	Nozzle Diameter (mm)	Infill Density (%)	Production Time for One Sample (min)
E-1	0.10	0.4	20	100
E-2	0.10	0.6	40	166
E-3	0.10	0.8	60	220
E-4	0.15	0.4	40	130
E-5	0.15	0.6	60	139
E-6	0.15	0.8	20	57
E-7	0.20	0.4	60	144
E-8	0.20	0.6	20	47
E-9	0.20	0.8	40	68

**Table 3:** MFPM and AEPM results for compression and LVI tests.

Experiment Number	Compression Tests			LVI Tests		
	Maximum Compressive Force (N)	Masses of Compression Test Samples (g)	MFPM (N/g)	Absorbed Energy (J)	Masses of LVI Test Samples (g)	AEPM (J/g)
E-1	13,541.41	12.00	1128.45	21.14	12.09	1.75
E-2	31,098.77	24.48	1270.37	43.26	24.04	1.79
E-3	58,942.78	36.51	1614.43	49.69	36.33	1.36
E-4	28,294.38	23.03	1228.59	34.28	23.27	1.47
E-5	59,791.54	36.47	1639.47	55.31	35.93	1.53
E-6	9084.16	12.26	740.96	18.24	12.00	1.52
E-7	47,510.42	34.23	1387.98	54.14	33.70	1.60
E-8	5818.77	12.15	478.91	31.82	11.89	2.67
E-9	32,492.65	24.20	1342.67	37.84	23.94	1.58

**Table 4:** Response table of S/N ratios for compressive and LVI tests.

Level	Compression Tests			LVI Tests		
	(A) Layer Thickness	(B) Nozzle Diameter	(C) Infill Density	(A) Layer Thickness	(B) Nozzle Diameter	(C) Infill Density
1	62.43	61.90	57.35	4.227	4.114	5.682
2	61.16	59.99	62.14	3.584	5.801	4.149
3	59.67	61.37	63.77	5.549	3.445	3.529
Delta	2.760	1.900	6.420	1.966	2.355	2.153
Σ Delta	11.080			6.474		
Weight (w_i_)	63.12%			36.88%		
Rank	2	3	1	3	1	2

**Table 5:** ANOVA results for compression tests.

Parameters	DF	SS	MS	F	*p*	Contribution (%)
Layer thickness (mm)	2	107,656	53,828	0.85	0.539	9.26
Nozzle diameter (mm)	2	24,977	12,488	0.20	0.835	2.15
Infill density (%)	2	903,419	451,710	7.17	0.122	77.74
Error	2	125,990	62,995			10.84
Total	8	1,162,041				100.00
S			250.98			
R-sq			89.06%			

**Table 6:** ANOVA results for LVI tests.

Parameters	DF	SS	MS	F	*p*	Contribution (%)
Layer thickness (mm)	2	0.31296	0.15648	3.65	0.215	25.90
Nozzle diameter (mm)	2	0.43668	0.21834	5.09	0.164	36.14
Infill density (%)	2	0.37299	0.18649	4.34	0.187	30.86
Error	2	0.08584	0.04292			7.10
Total	8	1.20847				100.00
S			0.207			
R-sq			92.90%			

**Table 7:** GRA results.

Experiment Number	Normalized Results		Grey Relation Coefficient (GRC)		Grey Relation Grade (GRG)	Rank
	MFPM	AEPM	MFPM	AEPM		
E-1	0.5597	0.2977	0.5317	0.4159	0.4890	8
E-2	0.6820	0.3282	0.6112	0.4267	0.5432	6
E-3	0.9784	0.0000	0.9586	0.3333	0.7280	2
E-4	0.6460	0.0840	0.5855	0.3531	0.4998	7
E-5	1.0000	0.1298	1.0000	0.3649	0.7658	1
E-6	0.2258	0.1221	0.3924	0.3629	0.3815	9
E-7	0.7833	0.1832	0.6976	0.3797	0.5804	3
E-8	0.0000	1.0000	0.3333	1.0000	0.5792	4
E-9	0.7443	0.1679	0.6616	0.3754	0.5560	5

**Table 8:** Response table for means for grey relation grade.

Level	(A) Layer Thickness	(B) Nozzle Diameter	(C) Infill Density
1	−4.757	−5.564	−6.442
2	−5.571	−4.121	−5.474
3	−4.856	−5.409	−3.267
Delta	0.813	1.534	3.176
Σ Delta	5.523		
Rank	3	2	1

## Data Availability

Data are contained within the article.

## References

[1] Tanveer Q., Mishra G., Mishra S., Sharma R. (2022). Effect of Infill Pattern and Infill Density on Mechanical Behaviour of FDM 3D Printed Parts—A Current Review. Mater. Today Proc..

[2] Bhuvanesh Kumar M., Sathiya P. (2021). Methods and Materials for Additive Manufacturing: A Critical Review on Advancements and Challenges. Thin-Walled Struct..

[3] Ngo T.D., Kashani A., Imbalzano G., Nguyen K.T.Q., Hui D. (2018). Additive Manufacturing (3D Printing): A Review of Materials, Methods, Applications and Challenges. Compos. Part B Eng..

[4] Rodríguez-Panes A., Claver J., Camacho A.M. (2018). The Influence of Manufacturing Parameters on the Mechanical Behaviour of PLA and ABS Pieces Manufactured by FDM: A Comparative Analysis. Materials.

[5] Mishra A.K., Chavan H., Kumar A. (2021). Effect of Material Variation on the Uniaxial Compression Behavior of FDM Manufactured Polymeric TPMS Lattice Materials. Mater. Today Proc..

[6] Öteyaka M.Ö., Çakir F.H., Sofuoğlu M.A. (2022). Effect of Infill Pattern and Ratio on the Flexural and Vibration Damping Characteristics of FDM Printed PLA Specimens. Mater. Today Commun..

[7] Tezel T., Ozenc M., Kovan V. (2021). Impact Properties of 3D-Printed Engineering Polymers. Mater. Today Commun..

[8] Peng C., Tran P., Nguyen-Xuan H., Ferreira A.J.M. (2020). Mechanical Performance and Fatigue Life Prediction of Lattice Structures: Parametric Computational Approach. Compos. Struct..

[9] Jap N.S.F., Pearce G.M., Hellier A.K., Russell N., Parr W.C., Walsh W.R. (2019). The Effect of Raster Orientation on the Static and Fatigue Properties of Filament Deposited ABS Polymer. Int. J. Fatigue.

[10] Dogan O. (2022). Short-Term Creep Behaviour of Different Polymers Used in Additive Manufacturing under Different Thermal and Loading Conditions. Stroj. Vestnik J. Mech. Eng..

[11] Karamooz Ravari M.R., Kadkhodaei M., Badrossamay M., Rezaei R. (2014). Numerical Investigation on Mechanical Properties of Cellular Lattice Structures Fabricated by Fused Deposition Modeling. Int. J. Mech. Sci..

[12] Yu B., Chen G., Sun J., Hua W., Wu W., Jin Y., Zhou W., Liu J., Zheng W. (2024). Finite Element Analysis of Warping and Mechanical Properties of 3D Parts Printed by Fused Deposition Modeling. J. Mater. Eng. Perform..

[13] Pernet B., Nagel J.K., Zhang H. (2022). Compressive Strength Assessment of 3D Printing Infill Patterns. Proceedings of the 29th CIRP Life Cycle Engineering Conference.

[14] Nace S.E., Tiernan J., Holland D., Ni Annaidh A. (2021). A Comparative Analysis of the Compression Characteristics of a Thermoplastic Polyurethane 3D Printed in Four Infill Patterns for Comfort Applications. Rapid Prototyp. J..

[15] Qin D., Sang L., Zhang Z., Lai S., Zhao Y. (2022). Compression Performance and Deformation Behavior of 3D-Printed PLA-Based Lattice Structures. Polymers.

[16] Silva R.G., Estay C.S., Pavez G.M., Viñuela J.Z., Torres M.J. (2021). Influence of Geometric and Manufacturing Parameters on the Compressive Behavior of 3d Printed Polymer Lattice Structures. Materials.

[17] Kamer M.S., Dogan O. (2024). Effects of Infill Pattern and Compression Axis on the Compressive Strength of the 3D-Printed Cubic Samples. Mater. Test..

[18] Ma Q., Rejab M.R.M., Kumar A.P., Fu H., Kumar N.M., Tang J. (2021). Effect of Infill Pattern, Density and Material Type of 3D Printed Cubic Structure under Quasi-Static Loading. Proc. Inst. Mech. Eng. Part C J. Mech. Eng. Sci..

[19] Aloyaydi B., Sivasankaran S., Mustafa A. (2020). Investigation of Infill-Patterns on Mechanical Response of 3D Printed Poly-Lactic-Acid. Polym. Test..

[20] Smardzewski J., Maslej M., Wojciechowski K.W. (2021). Compression and Low Velocity Impact Response of Wood-Based Sandwich Panels with Auxetic Lattice Core. Eur. J. Wood Wood Prod..

[21] Ye W., Cheng Y., Dou H., Zhang D., Yang F., Li Z., Cai W. (2023). Low-Velocity Impact Response and Compression Behaviour after the Impact of 3D-Printed CCFR Self-Sensing Honeycomb Structures. Compos. Part B Eng..

[22] Kaveloğlu S., Temiz Ş. (2024). Investigation of Low-Velocity Impact Performances of Sandwich Composites Manufactured Using 3d Printer. J. Fac. Eng. Archit. Gazi Univ..

[23] Wang S., Ma Y., Deng Z., Zhang S., Cai J. (2020). Effects of Fused Deposition Modeling Process Parameters on Tensile, Dynamic Mechanical Properties of 3D Printed Polylactic Acid Materials. Polym. Test..

[24] Tang C., Liu J., Yang Y., Liu Y., Jiang S., Hao W. (2020). Effect of Process Parameters on Mechanical Properties of 3D Printed PLA Lattice Structures. Compos. Part C Open Access.

[25] Chacón J.M., Caminero M.A., García-Plaza E., Núñez P.J. (2017). Additive Manufacturing of PLA Structures Using Fused Deposition Modelling: Effect of Process Parameters on Mechanical Properties and Their Optimal Selection. Mater. Des..

[26] Hsueh M.H., Lai C.J., Wang S.H., Zeng Y.S., Hsieh C.H., Pan C.Y., Huang W.C. (2021). Effect of Printing Parameters on the Thermal and Mechanical Properties of 3D-Printed PLA and PETG, Using Fused Deposition Modeling. Polymers.

[27] Farazin A., Mohammadimehr M. (2022). Effect of Different Parameters on the Tensile Properties of Printed Polylactic Acid Samples by FDM: Experimental Design Tested with MDs Simulation. Int. J. Adv. Manuf. Technol..

[28] Abbas T.F., Mansor K.K., Ali H.B. (2022). The Effect of FDM Process Parameters on the Compressive Property of ABS Prints. J. Hunan Univ. Nat. Sci..

[29] Rajpurohit S.R., Dave H.K. (2021). Impact Strength of 3D Printed PLA Using Open Source FFF-Based 3D Printer. Prog. Addit. Manuf..

[30] Irez A.B., Bilgen Bagci M. (2024). Effect of Printing Parameters on Impact Energy Absorption of Additively Manufactured Hierarchical Structures. Rapid Prototyp. J..

[31] (2016). Standard Test Method for Flatwise Compressive Properties of Sandwich Cores.

[32] (2015). Standard Test Method for Measuring the Damage Resistance of a Fiber-Reinforced Polymer Matrix Composite to a Drop-Weight Impact Event.

[33] Yuce B.E., Oral F. (2024). Multi Objective Optimization of Emission and Performance Characteristics in a Spark Ignition Engine with a Novel Hydrogen Generator. Energy.

[34] Tunçel O. (2024). Optimization of Charpy Impact Strength of Tough PLA Samples Produced by 3D Printing Using the Taguchi Method. Polymers.

[35] Yuce B.E., Nielsen P.V., Wargocki P. (2022). The Use of Taguchi, ANOVA, and GRA Methods to Optimize CFD Analyses of Ventilation Performance in Buildings. Build. Environ..

[36] Canbolat A.S., Bademlioglu A.H., Arslanoglu N., Kaynakli O. (2019). Performance Optimization of Absorption Refrigeration Systems Using Taguchi, ANOVA and Grey Relational Analysis Methods. J. Clean. Prod..

[37] Tunçel O., Tüfekci K., Kahya Ç. (2024). Multi-Objective Optimization of 3D Printing Process Parameters Using Gray-Based Taguchi for Composite PLA Parts. Polym. Compos..

[38] Yuce B.E. (2024). Optimization of Critical System Outputs of the Stirling Cycle Using Taguchi-Based Grey Relational Analysis. Proc. Inst. Mech. Eng. Part E J. Process Mech. Eng..

